# Pushing the Limits: Cognitive, Affective, and Neural Plasticity Revealed by an Intensive Multifaceted Intervention

**DOI:** 10.3389/fnhum.2016.00117

**Published:** 2016-03-18

**Authors:** Michael D. Mrazek, Benjamin W. Mooneyham, Kaita L. Mrazek, Jonathan W. Schooler

**Affiliations:** Department of Psychological and Brain Sciences, University of California Santa BarbaraSanta Barbara, CA, USA

**Keywords:** neuroplasticity, default network, executive network, insula, somatosensory cortex, mindfulness, working memory, well-being

## Abstract

Scientific understanding of how much the adult brain can be shaped by experience requires examination of how multiple influences combine to elicit cognitive, affective, and neural plasticity. Using an intensive multifaceted intervention, we discovered that substantial and enduring improvements can occur in parallel across multiple cognitive and neuroimaging measures in healthy young adults. The intervention elicited substantial improvements in physical health, working memory, standardized test performance, mood, self-esteem, self-efficacy, mindfulness, and life satisfaction. Improvements in mindfulness were associated with increased degree centrality of the insula, greater functional connectivity between insula and somatosensory cortex, and reduced functional connectivity between posterior cingulate cortex (PCC) and somatosensory cortex. Improvements in working memory and reading comprehension were associated with increased degree centrality of a region within the middle temporal gyrus (MTG) that was extensively and predominately integrated with the executive control network. The scope and magnitude of the observed improvements represent the most extensive demonstration to date of the considerable human capacity for change. These findings point to higher limits for rapid and concurrent cognitive, affective, and neural plasticity than is widely assumed.

## Introduction

The psychological and brain sciences have historically underestimated the human capacity for change. Recent discoveries have overturned the once widespread misconceptions that adults cannot generate new neurons (Gage, 2002), that individuals’ levels of happiness are unchanging (Diener et al., 2006), and that cognitive abilities like intelligence and working memory capacity (WMC) are fixed (Jaeggi et al., 2008). Yet current scientific understanding may still significantly underestimate the human capacity for change and the degree to which changes in numerous physiological and psychological capacities can occur in parallel. This misappraisal stems from the central logic of experimental design, which is to isolate the effect of a targeted manipulation through either control or randomization of all extraneous factors. Although this approach has indisputable value in establishing detailed casual relationships, it also leads to the study of variables in relative isolation. Given that most phenomena are the result of many interacting causes, there is a risk of neglecting how multiple influences combine to have greater effects than when they are studied in isolation.

Plasticity often occurs when there is a discrepancy between supply and demand (Lövdén et al., 2010). Much as muscles strengthen from repeated exertion, psychological capacities improve from progressively greater challenge (Lövdén et al., 2010). Discrepancy can also occur between existing beliefs and new information, as when beliefs about fixed personal capacities are challenged by evidence that these capacities can be improved through practice (Dweck, 2006). Numerous circumstances that elicit change can occur simultaneously, so plasticity should be able to occur in parallel across a wide variety of psychological and physiological systems. A change in one system may even become the precipitating cause for change in another.

Research from a variety of disciplines does illustrate the effectiveness of eliciting change through multifaceted interventions. The combination of exercise, nutrition, stress management, and social support can reverse coronary heart disease (Ornish et al., 1998). A wide diversity of psychological interventions are also multifaceted, including many forms of prevention and psychotherapy (Mrazek and Haggerty, 1994). Yet despite the effectiveness and widespread adoption of many relatively multifaceted interventions, existing approaches still target only a small proportion of all relevant factors and assess only a narrow set of outcomes. Furthermore, although highly multifaceted interventions could in principle be uniquely effective at revealing the human capacity for plasticity, the results of a recent meta-analysis suggest that interventions are generally most effective when they target no more than two to three behavior domains (e.g., diet and exercise) because this increases the likelihood that participants have sufficient motivation to comply with the recommend changes (Wilson et al., 2015). An outstanding question of both practical and theoretical importance is the extent to which a multifaceted intervention that is sufficiently motivating and effective can elicit substantial improvements across numerous psychological and physiological capacities. In short, it remains unknown what a highly intensive and comprehensive intervention might reveal about healthy adults’ capacity for concurrent cognitive, affective, and neural plasticity.

To examine the breadth and extent of plasticity that could be revealed by an intensive multifaceted intervention, we randomly assigned college undergraduates to either a 6-week training program or a waitlist control group. Participants in both conditions shared an interest and willingness to undergo an intensive training program. A large set of interdisciplinary outcome measures was necessary to characterize the potentially broad effects of the intervention and to test the possibility that individuals could simultaneously improve across a diverse set of outcomes. Assessments conducted before, after, and 6-weeks following the intervention included measures of physical fitness, a lipid profile, cognitive tasks, validated questionnaires, and resting-state fMRI. Patterns of resting-state functional connectivity (rs-FC) predict individual differences in cognitive abilities and are thought to reflect the repeated history of co-activation of brain regions (Guerra-Carrillo et al., 2014). Training-induced changes in rs-FC often correlate with improvements in performance, indicating that rs-FC provides a meaningful window into the brain dynamics underlying experience-dependent plasticity (Guerra-Carrillo et al., 2014).

## Materials and Methods

### Overview of Methods

**Figure 1:**
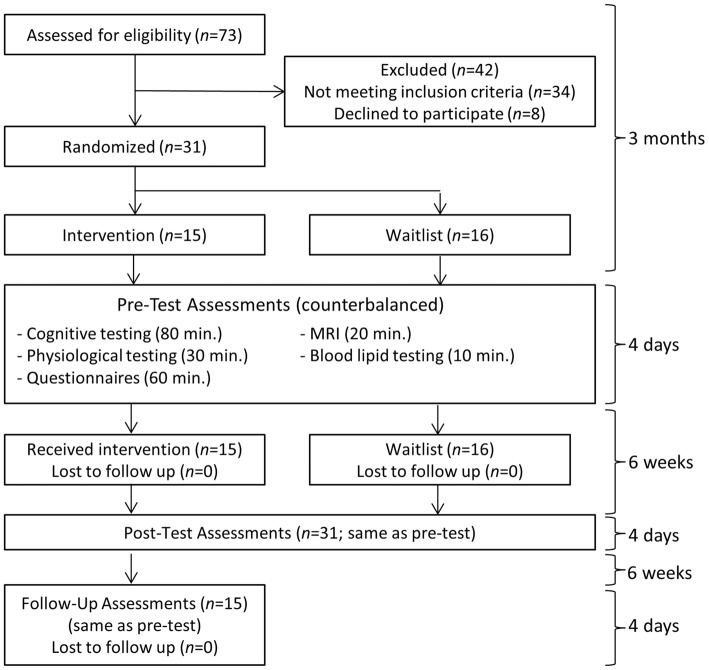
**Study overview**.

An overview of the study flow is provided in Figure 1. An estimated total sample size of 28 was needed to detect a between-group interaction from pre-test to post-test for a medium effect size (*f* = 0.25) with the conventional target of 80% power, two-tailed *p* < 0.05, two assessment points, and 0.60 test-retest measure reliability determined from a previous training study with shared outcome measures (computed with G*Power Software; Mrazek et al., 2013). Thirty-one college undergraduates (16 male and 15 female; mean age: 21.52 with SD: 2.20) from the University of California Santa Barbara were recruited to participate in what was described as an intensive lifestyle change program focused on exercise, nutrition, sleep, mindfulness, compassion, and relationships. The intervention (*N* = 15) and waitlist control (*N* = 16) conditions were balanced for age, gender, and college GPA using covariate adaptive randomization. Inclusion criteria were: (1) availability for all training and testing sessions; (2) a capacity to engage in physical exercise; and (3) no contraindications for MRI scanning. All applicants who met these criteria were included in the study. One more participant was enrolled in the waitlist control condition than in the intervention condition based on our expectation that at least one participant on the waitlist would drop out. Recruitment ended when the target sample size of 31 was reached. All 31 participants completed testing before and after the 6-week intervention. All 15 participants from the intervention condition returned for follow-up testing an additional 6 weeks later. With minor exceptions for temporary illness, all participants attended every session of the intervention.

The program was offered cost-free. Students received financial compensation at the rate of $10/h for the research assessments. Financial incentives for task performance were offered to participants based on their cumulative performance across tasks with objective measures (i.e., excluding all self-report, bloodwork, or MRI measures). This ensured that participants performed near their peak abilities at pre-testing, allowing for more a rigorous demonstration of improvements in participants’ actual capacities. The financial incentives also addressed alternative explanations based on task motivation. Participants were informed that an average payment for each of the three testing sessions would be approximately $25 but would vary based on their performance. Actual payments ranged between $20–30 depending on task performance.

Participants were tested in mixed groups by experimenters who were blind to condition. To test alternative explanations based on motivation effects or demand characteristics—which would predict an improvement across all measures—we included a measure of creativity that was not expected to be influenced by the intervention. We predicted improvements on all other measures except as specified below.

This research was approved by the University of California Santa Barbara’s Institutional Review Board and informed written consent was obtained from each participant at the beginning of the study. The consent procedure was approved by the Institutional Review Board.

### Intervention

The intervention convened for 5 h each weekday over a period of 6 weeks. Each day included 2.5 h of physical exercise, 1 h of formal mindfulness practice, and 1.5 h of lecture or discussion on topics related to sleep, nutrition, exercise, mindfulness, compassion, relationships, or well-being.

Each day began with 1 h of exercise in which participants completed a sequenced collection of exercises that targeted flexibility, balance, coordination, strength, and body awareness. Rate of respiration during these exercises was reduced to five breaths per minute by syncing the breath to subtle variations in music designed for this purpose.

Participants then completed 1 h of mindfulness practice. The majority of this time was spent engaging in focused attention meditation in which attention is directed to a single aspect of sensory experience (e.g., the physical sensations of breathing or walking). Participants also completed guided compassion meditation in which they deliberately generated feelings of compassion (wishing freedom from suffering) and kindness (wishing happiness) first toward themselves and then toward loved ones, acquaintances, and eventually strangers.

Each afternoon began with 1.5 h of lecture, discussion, or activity. Participants were introduced to key concepts and best practices in sleep, exercise, nutrition, alcohol consumption, mindfulness, gratitude, empathy, compassion, active listening, stress management, goal pursuit, and happiness.

Participants then completed 1.5 h of exercise under the direction of a fitness instructor with certifications in Pilates, yoga, and personal training. Workouts focused on Pilates twice a week, yoga once a week, and body weight circuit training once a week. A detailed workshop on anatomy and movement was offered once a week.

Throughout the program, participants were advised to limit alcohol intake to no more than one drink a day, to eat a diet of primarily whole foods, to restrict consumption of non-produce carbohydrates to after exercise, and to sleep 8–10 h each night while keeping a regular sleep schedule. One alcoholic drink was defined as 1.5 ounces of distilled spirits, 12 ounces of beer, or 5 ounces of wine.

Twice during the intervention, each participant met privately with an instructor for 20 min to discuss personal challenges and opportunities. Outside of the program, participants were advised to complete two high intensity interval training workouts each week and to engage in random acts of kindness each day. Participants kept a daily log of hours slept, alcoholic drinks consumed, workouts completed, and random acts of kindness. Each weekend, participants also kept a food log. These logs were reviewed by instructors and returned to participants each week with comments and suggestions for improvement. Following the 6-week intervention, participants received no additional instruction or support.

### Physiological Measures

Three of the most widely trained and studied components of physical fitness were assessed. Muscular flexibility was assessed with the Back-Saver Sit and Reach Test (Baltaci et al., 2003). With shoes removed, participants sat on the floor with one leg straight and the other leg bent. The foot of the straight leg was placed against a measurement box. Participants reached forward along the measurement box with hands placed on top of each other and palms facing down. Subjects repeated the test three times for each leg and an average score was computed. Cardiovascular fitness was assessed with an incremental max velocity treadmill test which alternated between 60 s of rest and exertion starting at 9 km/h on a 1% gradient and increasing 1 km/h each round of exertion until exhaustion (McNicol et al., 2009). Global core muscular endurance was assessed with the sport-specific endurance plank test (Tong et al., 2014). With forearms and toes on the ground, participants held their bodies in a straight plank until exhaustion.

Twelve hours fasted blood draws were conducted between 9 am and 11 am by the Student Health Clinic at the University of California Santa Barbara. Blood samples were shipped to Quest Diagnostics for assessment of high density lipoprotein (HDL cholesterol) and triglyceride levels. Low levels of HDL particles and high levels of triglycerides are associated with atherosclerosis within the walls of arteries and by extension, heart disease and stroke (Manninen et al., 1992). These risk factors for cardiovascular disease are among the most widely measured and studied physiological markers of health in medicine.

### Cognitive Measures

WMC is an intensively studied cognitive capacity that is highly predictive of an individual’s performance across a range of contexts (Unsworth et al., 2005). WMC was assessed via the widely used Operation Span Task (OSPAN), which presents 3–7 to-be-remembered stimuli in alternation with an unrelated processing task (i.e., verifying the accuracy of an equation). At the end of each trial, participants selected the presented items in the serial order in which they appeared. Stimuli for the OSPAN are chosen randomly from a list of letters and equations, ensuring that participants did not encounter the same pattern of stimuli from pre-testing to post-testing. Following standard procedure for this task, no participants had to be excluded for accuracy rates of less than 85% on the unrelated processing task (including errors caused by failing to respond within a response deadline based on latencies (M + 2.5 SDs) for 15 practice items). WMC was calculated as the proportion of total letters recalled in their correct position across all 15 trials. Total task duration was approximately 25 min.

Reading comprehension—a primary objective of educational systems and a central element of nearly all standardized testing—was assessed with a 20-min verbal reasoning section from the Graduate Record Examination (GRE) modified by excluding vocabulary-focused questions (Mrazek et al., 2013). GRE accuracy was calculated as the proportion of total questions answered correctly. There were three versions of the verbal GRE that were matched for difficulty and counterbalanced within each condition. Two non-native English speaking participants performed at chance and were excluded from analyses using the reading comprehension measure. Total task duration was approximately 25 min.

Mind-wandering—a shift of attention away from a task to unrelated concerns—is a pervasive mental state that is known to underlie performance on measures of working memory and reading comprehension (Mrazek et al., 2012). Mind-wandering during the OSPAN was measured with a widely used retrospective measure of task-unrelated thought following the OSPAN (Matthews et al., 1999). During the reading assessment, mind-wandering was measured with both thought sampling and instructions to self-catch instances of mind-wandering. Eight thought sampling probes occurred at unpredictable quasi-random intervals and asked participants to indicate to what extent their attention was either on-task or on task-unrelated concerns using a 1–5 Likert scale (1: completely on-task; 2: mostly on-task; 3: both on the task and unrelated concerns; 4: mostly on unrelated concerns; 5: completely on unrelated concerns). Participants also used a written form to count instances of mind-wandering that were self-caught independently of thought probes. A composite variable measuring task focus was computed from these three mind-wandering measures after standardizing all time-points with the mean and standard deviations from pre-testing. There is a broad literature validating self-report measures of mind-wandering obtained through thought-sampling using behavioral, event-related potential, and fMRI methodologies. This research cumulatively indicates that individuals are able to accurately report whether they have been mind-wandering—and even whether they have been aware of it—as revealed by distinct patterns of task performance and neural activation in association with self-reported mind-wandering (Smallwood and Schooler, 2015).

Creativity was assessed with the Remote Associates Test. Each of 15 trials presented three cue words that are all related to a fourth word (e.g., cream, skate, and water are all related to ice; Mednick and Mednick, 1967). Participants were given 45 s to solve each trial. Normative data was used to create three equally challenging versions of the test comprising questions of varying difficulty. Test version was counterbalanced within each condition. Given the association between mind-wandering and greater creativity (Baird et al., 2012), we predicted no improvement in creativity due to the intervention’s emphasis on using mindfulness training to develop a capacity for non-distraction and present-mindedness. The creativity measure was included to test alternative explanations based on motivation effects or demand characteristics, which would predict improvements across all measures. Total task duration was approximately 20 min.

Response inhibition was assessed with a flanker task derived from the Attention Network Task (Fan et al., 2002). Deliberately suppressing pre-potent responses is considered a central component of executive function (Miyake et al., 2000). However, debate exists regarding whether it draws on skills that are similar or distinct from those associated with the internal distraction we expected to be targeted by the intervention (Forster and Lavie, 2009; Smallwood, 2013). Each trial presented five arrows, and participants indicated whether a central arrow pointed left or right. The central arrow appeared either above or below fixation and was surrounded by flankers that pointed either in the same or opposite direction (half of trials had congruent flankers and the other half had incongruent flankers). After 24 practice trials, participants completed 144 trials. Response inhibition was calculated by subtracting the mean reaction time of all congruent flanking trials from the mean reaction time of incongruent flanking trials. Total task duration was approximately 10 min.

### Validated Scales

All the administered scales are widely used and have received extensive prior validation. Mindfulness was assessed with the *Mindful Attention and Awareness Scale*, a 12-item scale measuring attention to what is occurring in one’s present experience (e.g., “I find myself preoccupied with the future or the past”; reverse scored). Mind-wandering was assessed with the 5-item *Mind-Wandering Questionnaire* (e.g., “While reading, I find I haven’t been thinking about the text and must therefore read it again”). Mood was assessed using the *Positive and Negative Affect Schedule*. This measure consists of two 10-item scales measuring positive and negative affect. Participants were presented with words representing either positive or negative moods and asked to rate to what extent they felt a certain way over the last 2 weeks. Mood was calculated as a ratio of the positive to negative mood subscales. Self-esteem was assessed with the 10-item *Rosenberg Self-Esteem Scale* (e.g., “I feel that I am a person of worth, at least on an equal plane with others”). Self-efficacy was assessed using the *New General Self-Efficacy Scale* (e.g., “I believe I can succeed at most any endeavor to which I set my mind”). Stress was measured with the 4-item *Perceived Stress Scale* (e.g., “How often have you felt that you were unable to control the important things in your life?”). Life satisfaction was assessed using the 5-item *Satisfaction with Life Scale* (e.g., “In most ways my life is close to ideal”). Compassion was measured using the 5-item subscale of the *Positive Emotion Dispositions Scale* (e.g., “When I see someone hurt or in need, I feel a powerful urge to take care of them”).

### Statistical Analysis for Physiological, Cognitive, and Scale Measures

Data was analyzed using mixed model analysis of variance (ANOVA) to determine whether there was a statistically significant interaction between condition and testing session. Uncorrected follow-up paired-*t* tests were performed to interpret any interaction. To assess persistence of effects after the training was complete, paired sample *t*-tests compared participants baseline and 6-week follow up scores.

### MRI Methods and Analysis

#### MRI Acquisition and Data Processing

MRI images were obtained using a Siemens 3.0T Magnetom TIM TRIO (SYNGO MR B17) scanner. A high-resolution T1-weighted anatomical scan was acquired for each subject (MPRAGE, TR = 2300 ms; TE = 2.98 ms; TI = 900 ms; flip angle = 9°; FOV = 256 mm; acquisition voxel size = 1 × 1 × 1 mm), followed by a T2*-weighted echo-planar imaging (EPI) sequence eyes closed resting-state scan of the whole brain (TR = 2000 ms; TE = 30 ms; flip angle = 90°; acquisition matrix = 64 × 64; FOV = 192 mm; acquisition voxel size = 3 × 3 × 3 mm; 37 interleaved slices; 206 volumes).

#### Structural (T1) Data Processing

Cortical surface reconstruction was performed on T1 scans using FreeSurfer^1^. For each subject, nonlinear transformation from T1 to the 2 mm MNI152 template was calculated using Advanced Normalization Tools (ANTs)^2^.

#### Resting-state fMRI (EPI) Data Processing

The first four volumes of each EPI sequence were removed to eliminate potential effects of scanner instability. Slice timing of the EPI images was performed using AFNI’s 3dTshift, followed by motion correction of the images using AFNI’s 3dvolreg. Affine co-registration of the mean EPI image and T1 volume was then calculated using FreeSurfer’s BBRegister. Brain, cerebrospinal fluid (CSF), and white matter masks were extracted after FreeSurfer parcellation and transformed into EPI space using BBRegister (thresholded at 0.5 after interpolation). Co-registered EPI images were then masked using the brain mask. Principal components of physiological noise were estimated using CompCor (Behzadi et al., 2007), where a joined white matter and CSF mask and voxels of highest temporal variance were used to extract two sets of principal components (i.e., aCompCor and tCompCor); motion and intensity outliers in the EPI sequence were also discovered based on intensity and motion parameters using ArtDetect^3^. All timeseries were then denoised using a general linear model (GLM) model with the motion parameters, CompCorr components, and intensity outliers used as regressors (note that global signal has not been regressed). Finally, resultant timeseries were smoothed using FreeSurfer with 5 mm full width half minimum (FWHM) surface and volume kernels, highpass (0.01 Hz) and lowpass (0.1 Hz) filters were applied using FSL, and nonlinear normalization warping from subject functional/anatomical space to 2 mm MNI space was computed using ANTs, with each volume transformed into 2 mm MNI space using this transformation.

#### Independent Component Analysis (ICA) and Region-of-Interest Selection

Thirty subjects’ resting-state scans from the pre- and post-intervention scanning sessions were entered into Group ICA (gICA) implemented in GIFT^4^. Twenty independent components were extracted by independent components analysis (ICA) decomposition using the Infomax algorithm. The component containing the default mode network (DMN) was then identified by spatial correlation with a weighted template provided in GIFT (*r* = 0.251). The average component (across subjects and sessions) for the DMN was Fisher *r*-to-*z* transformed, where the value at each voxel represented the degree of correlation between the voxel and component timecourses. In order to identify the location that demonstrated the most highly correlated activity within the DMN, we applied a height threshold of *z* = 3 and a cluster-extent threshold of 100 voxels to the mean default mode component. This threshold revealed two bilateral clusters within the posterior cingulate cortex (PCC) with peak correlations at MNI: (−14, −58, 16) and (14, −54, 14).

#### Default Mode Network Functional Connectivity

Four millimetre-radius spheres were drawn around the peak activation coordinates for each PCC cluster. For all subjects at each scanning session, two seed timecourses were produced by averaging the timecourses of the voxels within each of the two spherical PCC masks. Full brain connectivity (correlation) maps were calculated for each seed region using AFNI, Fisher *r*-to-*z* transformed, and calculated in MNI152 space. These functional connectivity maps were averaged to provide a single bilateral connectivity map for the PCC to be used in subsequent analyses.

#### Degree Centrality

Degree-centrality is a graph-theory/network measure that can be calculated from functional MRI data. Treating each voxel as a node within a graph, the degree centrality of a node indexes the number of other nodes throughout the graph (i.e., brain) that exhibit activity correlated with the node in question. Regions with the highest degree centrality are considered to be those that are functionally connected with the most other regions. Due to their rich interconnectedness with other areas, these regions are often considered to be “hubs” of functional networks (Bullmore and Bassett, 2011). Degree centrality calculations were performed on MNI-transformed resting-state images using the degree centrality calculation within the FastECM algorithm (Wink et al., 2012). This computes for each scan the Pearson correlation values between individual voxel timecourses and provides a weighted measure of the connection strengths between a given voxel and all others. As such, no arbitrary criterion were used to determine whether functional connections were present between individual voxels (i.e., degree centrality maps were not binarized) but rather, for a particular voxel being assessed, each other voxel in the image contributed to the degree centrality of this voxel in accordance with the magnitude of the correlation between the two voxels. In order to eliminate any spurious effects of subject motion or variance in signal intensity in the calculation of degree centrality, the resting-state scans were intensity normalized as part of pre-processing. Motion parameters (six dimensions) and intensity- and motion-based outlier volumes were also regressed from the scanning data using GLM.

#### fMRI Statistical Analyses

One subject from the waitlist control condition had a frontal lobe structural abnormality and was excluded from all MRI analyses. Group-level analysis was conducted using the GLM framework implemented in SPM8 (Wellcome Trust Department of Imaging Neuroscience, University College London, UK). Whole-brain analyses employing ANOVA on data from randomized controlled trials carry a risk of producing findings that are not driven by the experimental manipulation but rather by a combination of chance baseline differences and chance divergence between conditions over time (Voss et al., 2010). This risk is reduced by using stringent corrections for multiple comparisons, but can be further minimized using a statistical procedure that firsts detects regions showing significant changes over time in the intervention condition and then subsequently examines these regions at pre-test and post-test across both conditions (Hölzel et al., 2011). We employed this approach and then examined the correlation of our rs-FC results with behavioral data to provide converging evidence that the neuroimaging findings reflected meaningful changes in brain function.

For both the degree centrality maps and the PCC-seeded functional connectivity maps, paired-*t* tests were performed comparing participants from the intervention condition across the pre-testing and post-testing scanning sessions. Whole-brain analyses were conducted, correcting for multiple comparisons using topological FDR (Chumbley et al., 2010). Cluster forming threshold was set at *p* < 0.001 and cluster size threshold was set at *p* < 0.05 (FDR corrected). Mean values for clusters reaching significance in the initial paired-*t* test were then extracted for all 30 subjects from both the pre-test and post-test scans. These values were subjected to a mixed model ANOVA to determine whether there was a statistically significant interaction between condition and testing session (*p* < 0.05). Finally, for clusters that demonstrated a significant interaction within the ANOVA, follow-up paired-*t* tests were performed to confirm that there was neither a difference in cluster values between the intervention and waitlist-control groups at pre-testing nor a difference within the waitlist-control group from pre-testing and post-testing. Two clusters from the degree centrality analysis revealed significant pre-testing differences (*p* < 0.05) and were not subjected to further analysis. Subsequently reported results passed all of the described criteria for statistical significance. A follow-up paired *t-*test within the intervention group was not necessary because each finding originated from a whole-brain paired* t*-test within the intervention condition that employed strict FDR correction for multiple comparisons.

The persistence of the neuroimaging effects was assessed at the 6-week follow-up. Degree centrality was calculated for each participant using same procedure described above. Functional connectivity maps for the PCC were calculated using the two seed regions obtained previously through ICA. Functional connectivity values at each voxel were averaged across both seeds to create a single connectivity map. Degree centrality and functional connectivity values were then extracted from the locations of the significant clusters from previous analyses and averaged across the voxels within each cluster. Paired-*t* tests compared the pre-test and 6-week follow-up values.

## Results

**Figure 2:**
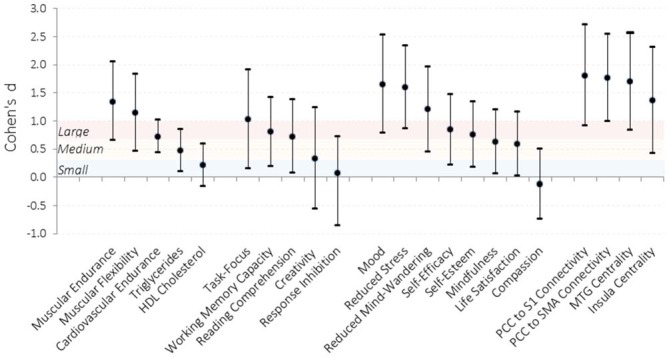
**Effect sizes of improvements observed in the intervention condition compared to the waitlist control condition.** Effect sizes for each dependent measure were calculated by computing the difference in change from pre-testing to post-testing for the intervention relative to the waitlist control and dividing by the pooled standard error across testing sessions. Error bars represent 95% confidence intervals. Shaded sections demarcate traditional effect size descriptions. PCC, posterior cingulate cortex; S1, somatosensory cortex; SMA, supplemental motor area; MTG, middle temporal gyrus. *N* = 31.

Relative to the waitlist control group that did not significantly change over the 6 weeks on any measure, the intervention led to a broad set of substantial improvements in physiology, cognition, and affect (Figure 2; Table 1). Participants demonstrated enhanced muscular endurance, cardiovascular endurance, muscular flexibility, and reduced triglyceride levels (a blood lipid associated with heart disease and diabetes). They also improved their task-focus, working memory capacity, and performance on a standardized test of reading comprehension derived from the GRE. Validated questionnaires revealed that participants experienced improved mood, life satisfaction, self-esteem, self-efficacy, and mindfulness, as well as reduced stress and mind-wandering. Despite these broad and substantial improvements, other measures were unaffected by the training. As predicted, there was no significant improvement in creativity. Response inhibition, HDL cholesterol, and self-reported compassion also did not significantly change.

**Table 1:** **Means and standard deviations (SDs) for pre-test and post-test assessments**.

	Intervention			Waitlist control		
	Pre-test	Post-test	Paired *t*-test	Pre-test	Post-test	Paired *t*-test	ANOVA	
Measure	*M (SD)*	*M (SD)*	*p*	*M (SD)*	*M (SD)*	*p*	*F*	*p*
Muscular endurance (s)	108.04 (71.87)	194.17 (92.81)	0.003	90.86 (45.23)	88.83 (45.29)	0.291	13.66	0.001
Muscular flexibility (cm)	22.40 (6.32)	30.00 (4.98)	0.000	22.88 (6.87)	23.74 (6.08)	0.350	15.29	0.001
Cardiovascular endurance (s)	288.40 (120.17)	376.52 (114.17)	0.000	327.88 (138.92)	319.37 (159.32)	0.449	33.98	0.000
Triglycerides (mg/dL)	93.14 (35.36)	68.21 (23.43)	0.007	98.07 (51.83)	107.67 (74.53)	0.470	5.63	0.025
HDL cholesterol (mg/dL)	54.71 (16.86)	59.00 (16.76)	0.058	51.60 (15.32)	52.53 (14.44)	0.828	1.26	0.271
Task focus (*z*-score)	0.17 (0.74)	0.64 (0.42)	0.040	−0.07 (0.54)	−0.19 (0.54)	0.438	5.51	0.026
Working memory (items)	42.73 (15.66)	53.60 (16.71)	0.020	54.13 (17.76)	52.13 (14.01)	0.443	7.27	0.012
Reading comprehension (%)	57.79 (15.54)	71.43 (20.32)	0.006	57.27 (23.00)	56.36 (24.17)	0.815	6.57	0.016
Creativity (correct answers)	5.80 (2.84)	7.53 (2.77)	0.113	5.81 (2.46)	6.75 (1.65)	0.240	0.50	0.486
Response inhibition (ms)	90.64 (40.13)	80.31 (43.74)	0.747	86.98 (33.45)	73.97 (30.34)	0.247	0.03	0.856
Mood (1–5 Likert)	0.99 (0.96)	2.24 (0.75)	0.001	1.18 (0.92)	0.838 (1.16)	0.056	20.22	0.000
Stress (1–5 Likert)	2.53 (0.63)	1.72 (0.56)	0.000	2.38 (0.53)	2.64 (0.89)	0.105	23.93	0.000
Mind-wandering (1–6 Likert)	4.27 (0.38)	3.29 (0.88)	0.001	3.98 (0.79)	3.99 (1.05)	0.952	10.60	0.003
Self-efficacy (1–5 Likert)	3.87 (0.49)	4.17 (0.46)	0.007	4.23 (0.47)	4.11 (0.61)	0.125	7.56	0.010
Self-esteem (1–4 Likert)	2.94 (0.48)	3.25 (0.38)	0.014	3.21 (0.58)	3.14 (0.60)	0.386	8.65	0.006
Mindfulness (1–6 Likert)	3.71 (0.49)	4.08 (0.75)	0.042	3.70 (0.78)	3.61 (0.78)	0.891	5.35	0.028
Life satisfaction (1–7 Likert)	4.93 (0.89)	5.73 (0.91)	0.004	4.69 (1.59)	4.75 (1.63)	0.758	6.02	0.020
Compassion (1–7 Likert)	5.67 (0.77)	5.71 (0.75)	0.921	5.59 (0.79)	5.74 (1.13)	0.467	0.14	0.281

*F and p values are computed from a mixed-model ANOVA with condition and pre-test/post-test as factors*.

**Figure 3:**
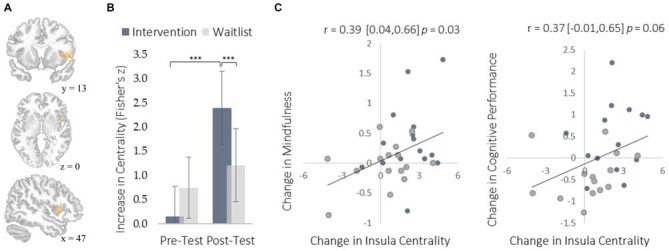
**Changes in degree centrality in the insula. (A)** The location of a significant cluster in the right insular cortex demonstrating increased degree centrality (MNI peak-coordinate: 48, 10, 4). **(B)** Mean degree centrality values within the insula cluster for the intervention and waitlist groups at pre-testing and post-testing. **(C)** Scatterplots correlating changes in insula degree centrality with changes in mindfulness and changes in cognitive performance from pre-testing to post-testing. Error bars and brackets represent 95% confidence intervals. ***p < 0.001. *N* = 30.

**Figure 4:**
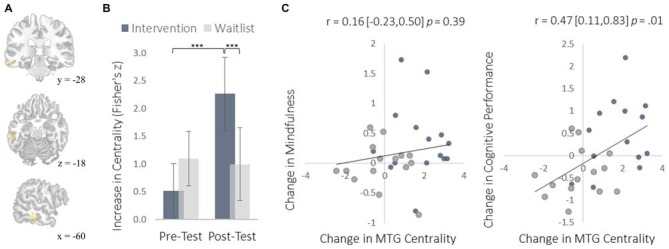
**Changes in degree centrality in the MTG. (A)** The location of significant clusters in the left MTG demonstrating increased degree centrality (MNI peak-coordinate: −60, −32, −16). **(B)** Mean degree centrality values within the MTG cluster for the intervention and waitlist groups at pre-testing and post-testing. **(C)** Scatterplots correlating changes in MTG degree centrality with changes in mindfulness and changes in cognitive performance from pre-testing to post-testing. Error bars and brackets represent 95% confidence intervals. ***p < 0.001. *N* = 30.

We examined neural plasticity using rs-FC, which examines the co-activation of functionally integrated brain regions (Guerra-Carrillo et al., 2014). Changes in rs-FC were assessed using two complementary methods. First, we used a data-driven approach to identify brain regions that showed the greatest changes in degree centrality, which measures the number of functional connections that each brain region has with all others. This approach detects regions exhibiting the largest changes in functional connectivity throughout the brain without requiring *a priori* specification of particular regions of interest. The intervention resulted in greater degree centrality within the right insula (Figure 3) and the left middle temporal gyrus (MTG; Figure 4).

**Figure 5:**
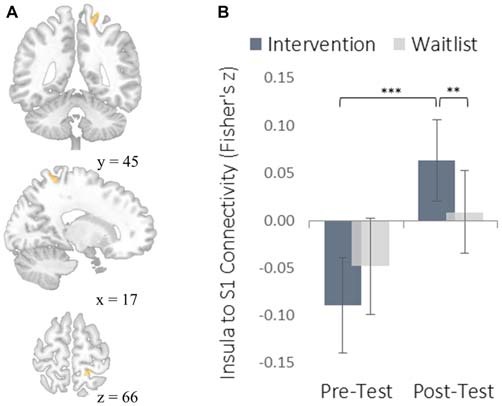
**Functional connectivity results using the degree-centrality-based insula seed region. (A)** The location of a significant cluster in the right somatosensory cortex demonstrating increased functional connectivity with the insula seed; MNI peak-coordinate: (46, −20, 20). Cluster forming threshold set at *p* < 0.001 and cluster size threshold set at *p* < 0.05 (FDR corrected). **(B)** Mean Fisher *r-*to-*z* transformed functional connectivity values for the intervention and waitlist groups at pre-testing and post-testing. **p < 0.01, ***p < 0.001. Error bars represent 95% confidence intervals.

The insula plays a well-established role in bodily and emotional awareness (Craig, 2011). Follow-up functional connectivity analyses using the insula as a seed region revealed that training increased connectivity between the insula and somatosensory cortex, a region involved in sensation and bodily awareness (Figure 5). Increases in degree centrality of the insula were positively correlated with improved dispositional mindfulness, providing converging support for each result and suggesting that these changes in rs-FC may underlie an increased capacity for present moment awareness.

**Figure 6:**
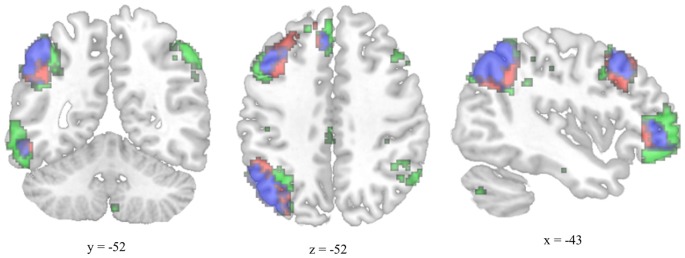
**Overlap between the functional connectivity map of the degree-centrality-based MTG cluster and the left executive control network.** The left executive control network was mapped using a template from Shirer et al. (2012). The functional connectivity map for the left-hemisphere MTG cluster was generated using pre-test resting-state images from all participants and thresholded for viewing purposes using a cluster forming threshold set at *p* < 0.001 and a cluster size threshold set at *p* < 0.05 (FDR corrected). Executive control network template is green, MTG connectivity map is red, and overlap is blue.

The MTG cluster that increased in degree centrality was located within the executive control network—a collection of brain regions involved in regulating cognitive processes. A confirmatory connectivity analysis using the pre-intervention data from all participants confirmed that the MTG cluster was extensively and primarily integrated with numerous regions comprising the executive control network (Figure 6). Changes in degree centrality of the MTG were positively correlated with changes in cognitive performance (a composite measure of WMC and reading comprehension; Figure 4). This indicates that changes in rs-FC of a brain region within the executive control network predicted improvements in laboratory tasks that require executive control. A follow-up seed-based analysis for the MTG revealed no specific regions with which the MTG showed particularly increased rs-FC.

**Figure 7:**
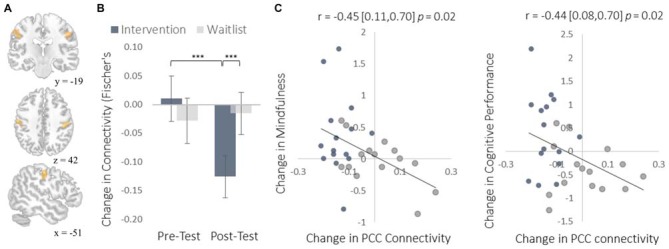
**Results for somatosensory cortex (S1) regions showing a decrease in functional connectivity with the PCC seed regions of interest (ROIs). (A)** The location of S1 functional connectivity changes, comprised of bilateral S1 clusters; left-hemisphere cluster MNI peak-coordinate: (−52, −16, 40); right-hemisphere cluster MNI peak-coordinate: (50, −18, 38). Subsequent reports integrate these two regions into one set of S1 regions by computing the mean functional connectivity value across all voxels within both clusters. **(B)** Mean Fisher *r*-to-*z* transformed functional connectivity values. **(C)** Scatterplots correlating changes in PCC-S1 functional connectivity with changes in mindfulness and changes in cognitive performance from pre-testing to post-testing. Error bars and brackets represent 95% confidence intervals. ***p < 0.001. *N* = 30.

**Figure 8:**
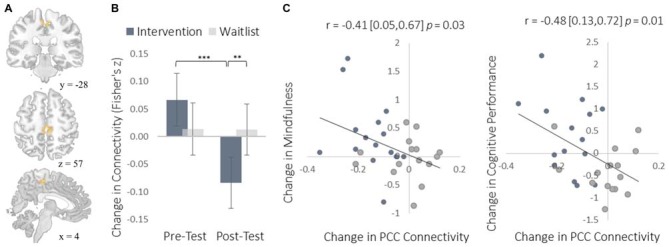
**Results for the SMA cluster showing a decrease in functional connectivity with the PCC seed ROIs. (A)** The location of the SMA cluster; MNI peak-coordinate: (4, −26, 52). Cluster forming threshold set at *p* < 0.001 and cluster size threshold set at *p* < 0.05 (FDR corrected). **(B)** Mean Fisher *r-to-z* transformed functional connectivity values for the intervention and waitlist groups at pre- and post-testing. **(C)** Scatterplots correlating changes in PCC-SMA functional connectivity with changes in mindfulness and changes in cognitive performance from pre-testing to post-testing. SMA, supplemental motor area; PCC, posterior cingulate cortex. Error bars and brackets represent 95% confidence intervals. **p < 0.01, ***p < 0.001.

To complement the data-driven degree centrality results, we used a seed-based approach to examine changes in the rs-FC of the DMN. The DMN is a collection of brain regions that are involved in internally directed thought and mind-wandering (Christoff et al., 2009; Andrews-Hanna et al., 2014). Given the intervention’s emphasis on mindfulness, we predicted that the DMN would show changes in rs-FC at rest that would correspond to a shift from discursive thought toward present moment awareness. To select a seed region, we applied ICA to identify the DMN and then applied a statistical threshold to this component to identify the most strongly integrated region. This resulted in two regions located bilaterally in the PCC, which is widely established as a central hub of the DMN (Andrews-Hanna et al., 2010). The intervention led to reduced rs-FC between these PCC regions and both the somatosensory cortex (Figure 7) and the supplemental motor area (SMA; Figure 8). These reductions in rs-FC were correlated with increased dispositional mindfulness and enhanced cognitive performance.

Although participants received no support or instruction following the intervention, the effects of training persisted for an additional 6 weeks (Table 2). Relative to their initial baseline, participants continued to show improved muscular endurance, cardiovascular endurance, muscular flexibility, reading comprehension, and WMC. They also continued to report higher levels of mood, life satisfaction, self-esteem, self-efficacy, and mindfulness, as well as reduced stress and mind-wandering. The insula and MTG showed trends toward increased degree centrality, while rs-FC between the DMN and somatosensory cortex was still significantly reduced.

**Table 2:** **Persistence of training effects from baseline to 6-week follow-up**.

Measure	Mean difference	SD of mean difference	95% CI of mean difference		Cohen’s *d*	Paired-*t* *t* value	Paired-*t* *p* value
Muscular endurance (s)	44.78	59.44	11.86	77.70	0.70	2.92	0.011
Muscular flexibility (cm)	7.28	5.70	4.13	10.44	1.20	4.95	0.000
Cardiovascular endurance (s)	44.01	62.05	8.18	79.84	0.33	2.65	0.020
Task focus (*z*-score)	0.61	0.71	0.20	1.02	1.09	3.20	0.007
Working memory (items)	8.87	12.21	2.10	15.63	0.55	2.81	0.014
Reading comprehension (% score)	10.39	17.32	0.39	20.39	0.50	2.24	0.043
Creativity (correct answers)	0.86	2.45	−0.55	2.27	0.35	1.31	0.212
Response inhibition (ms)	−27.24	27.86	−42.67	−11.81	0.74	3.79	0.002
Mood (1–5 Likert)	0.89	1.25	0.20	1.59	0.94	2.77	0.015
Stress (1–5 Likert)	−0.52	0.74	−0.93	−0.11	0.80	2.70	0.017
Trait mind-wandering (1–6 Likert)	−0.97	0.82	−1.34	−0.52	1.25	4.60	0.000
Self-efficacy (1–5 Likert)	0.38	0.32	0.21	0.56	0.75	4.66	0.000
Self-esteem (1–4 Likert)	0.27	0.34	0.09	0.46	0.53	3.14	0.007
Mindfulness (1–6 Likert)	0.49	0.44	0.25	0.73	0.70	4.33	0.001
Life satisfaction (1–7 Likert)	0.67	0.78	0.23	1.10	0.53	3.29	0.005
Compassion (1–7 Likert)	0.27	0.52	−0.03	0.56	0.31	1.96	0.070
PCC to S1 connectivity (Fisher’s *z)*	−0.05	0.06	−0.08	−0.01	0.57	2.80	0.014
PCC to SMA connectivity (Fisher’s *z*)	−0.04	0.13	−0.11	0.04	0.42	1.08	0.298
MTG degree centrality (Fisher’s *z*)	0.81	1.90	−0.25	1.86	0.69	1.64	0.124
Insula degree centrality (Fisher’s *z*)	0.61	1.44	−0.19	1.41	0.45	1.64	0.123

*To allow for direct comparison to Figure 1, effect sizes were calculated as the mean difference divided by the pooled standard deviation from pre-testing and post-testing. PCC, posterior cingulate cortex; S1, somatosensory cortex; SMA, supplemental motor area; MTG, middle temporal gyrus*.

## Discussion

These findings cumulatively indicate that a multifaceted intervention can simultaneously produce substantial and enduring improvements across a wide variety of psychological and physiological systems in healthy young adults. The magnitude of the effect sizes indicate considerably greater changes than observed from more narrowly-focused interventions, including improvements in mood and stress that were more than 2.5 times greater than typically observed from mindfulness training alone (Grossman et al., 2004; Eberth and Sedlmeier, 2012). These findings provide an important exception to the prevailing view that highly multifaceted interventions are less effective (Wilson et al., 2015). The present results indicate that multifaceted interventions that are effectively designed and sufficiently motivating can elicit large and diverse improvements that reveal the substantial adult capacity for cognitive, affective, and neural plasticity.

The increased degree centrality detected within the insula suggests heightened interoceptive awareness of bodily sensations, particularly given the increase in rs-FC between the insula and primary somatosensory cortex (S1). Whereas S1 became more functionally integrated with the insula, it became less integrated with the PCC. As a key hub of the DMN, the PCC is involved in mind-wandering and elaborative conceptual thought (Christoff et al., 2009; Andrews-Hanna et al., 2014). The decrease in rs-FC between the PCC and S1 may represent less conceptual elaboration of sensory experience (Farb et al., 2007). Furthermore, the functional decoupling between the PCC and SMA may have been associated with a reduction in the degree to which elaborative internal speech processes were engaged as a result of DMN activity during the resting-state, as SMA activity has previously been associated with internal speech mechanisms (Ryding et al., 1996). Considered altogether, these results suggest a mechanistic account of changes in rs-FC that underlie an improved capacity for non-distraction from one’s immediate experience—a skill that underlies cognitive performance on tasks requiring continuous attention and that was improved markedly by the intervention (Mrazek et al., 2012).

We observed instances in which the intervention altered the functional connectivity of two brain regions from a negative to a positive correlation (insula-S1), a neutral to a negative correlation (PCC-S1), and from a positive to a negative correlation (PCC-SMA). A negative correlation between two brain regions indicates that increases in the activation of one region tend to be associated with a decrease in activation of another. Direct inhibition of one region by the other is one possible explanation for this relationship. An inhibitory relationship mediated by yet another brain region is also possible. A third possibility is that the activations of two regions are diverging due to the current psychological processes being engaged and not because of any inhibitory function. For example, consider the FC between PCC and SMA/S1. At pre-testing there was some indication of positive FC between the PCC and SMA, which could indicate a default tendency to conceptually elaborate sensory experience: when a salient sensory experience occurs, there is a concomitant increase in conceptual elaboration (Farb et al., 2007). The intervention reversed this relationship such that there was negative FC between PCC and both S1 and SMA. Given that this reversal correlated with increases in mindfulness, the negative FC at post-testing may indicate that salient sensory experiences drew attention to those perceptual experiences (increasing S1 and SMA activation) and away from their conceptual elaboration (decreasing PCC activation). This interpretation—which does not specify any specific inhibitory relationship—is consistent with the training induced increase in FC between insula and S1, which may indicate greater monitoring of sensory experiences. By this view, the negative FC between insula and S1 at pre-testing may represent a low prioritization of attention to sensory experience. This interpretation will need to be reexamined in light of future research, particularly with respect to studies using effective connectivity measures like Granger causality to assess any inhibitory relationships between these regions.

Research into the plasticity of cognitive abilities like WMC often involve extensive practice of a training task followed by an attempt to demonstrate a transfer of improvement beyond the trained task to an unpracticed task that measures the same ability (near transfer) or a related but distinct ability (far transfer). Demonstrating transfer effects is crucial for ruling out explanations based on task-specific learning or strategies, yet ongoing debate exists regarding the transfer of working memory training (Klingberg, 2010; Melby-Lervåg and Hulme, 2013; Redick et al., 2013). The strongest evidence for plasticity of a cognitive ability is therefore derived from a training task with little resemblance to the outcome measure. The present intervention improved both WMC and reading comprehension despite involving no practice of training tasks for these abilities, thereby providing a rigorous demonstration of cognitive plasticity that cannot be attributed to overlap between training and testing contexts.

The interpretation of any scientific finding hinges on its susceptibility to confounds and alternative explanations, and careful consideration is warranted in research that does not utilize the traditional approach of carefully isolating a targeted manipulation. Yet interventions across a wide range of disciplines rarely succeed in producing a truly single-faceted manipulation. This alone does not threaten internal validity if the intervention is examined as a whole, though it does constrain specific conclusions about the most potent elements of the intervention. As long as conclusions are limited to the intervention’s overall effect, multifaceted interventions pose no greater challenge for interpretation than their more narrowly focused counterparts.

Whether narrow or broad in focus, all intervention studies face a similar set of methodological challenges. We sought to address alternative explanations for our findings through numerous methodological controls. Participants were tested in mixed groups by experimenters who were blind to condition, thereby minimizing expectancy effects arising from unconscious experimenter bias. Additionally, the inclusion of resting state neuroimaging and physiological measures provide compelling evidence of changes that are not susceptible to motivation effects or demand characteristics. Importantly, changes in resting state functional connectivity correlated with changes in behavioral and subjective measures, lending greater certainty that the latter changes represent genuine improvements. Furthermore, the absence of improvements in self-reported compassion—an explicit focus of the intervention—suggests the observed findings are not due to demand characteristics.

Although a waitlist control condition is not well-suited for all research programs, it is the most appropriate choice for an investigation that explores the limits of cognitive, affective, and neural plasticity using an intensive and multifaceted intervention. A waitlist control effectively addresses effects due to developmental maturation, repeated exposure to assessments, and self-selection of participants based on pre-existing characteristics. When combined with strategies to control for motivation effects (e.g., financial incentives at pre-test and post-test), a waitlist condition controls for only those factors that are unrelated to the intervention and therefore provides the best estimate of the effect size of the intervention as a whole. Given that the theoretical underpinning of this investigation was to explore how multiple influences combine to facilitate change, effects due to expectation of improvement or therapeutic alliance—which are sometimes considered confounding effects—represent meaningful elements of the intervention. It would not only be impossible to create an active control condition precisely matched in participants’ expectations of change or interaction with an effective teacher, but doing so would misguidedly attempt to control for a meaningful element of the intervention and thereby bias the effect size estimate.

An inevitable limitation of this study—and any investigation that utilizes an even modestly multifaceted intervention—is the inability to specify which aspects of the intervention produced the observed effects and by what mechanism. Tightly controlled experiments are therefore crucial even though they necessarily neglect the complex interactions that often underlie the process of change in human life. The complementary nature of multifaceted and narrow interventions will ultimately provide the most complete understanding of cognitive, affective, and neural plasticity.

Future research could improve and extend this work in several ways. It is well-established that the extent of plasticity varies across the lifespan (Baltes, 1987; Brehmer et al., 2007). The effects of similarly modeled interventions might have especially large and enduring effects among youth given the considerable plasticity at this stage of development, though older adults may also benefit if multifaceted training programs can prevent cognitive decline. Future research could also examine a wide variety of additional outcomes, including structural changes within the brain that can emerge within only weeks of training (Draganski et al., 2004; Tang et al., 2010). Studies with larger sample sizes could also identify factors that predispose individuals to benefit from an intervention. Finally, future research should strive to identify the optimal number of distinct program elements that will still allow individuals sufficient time and motivation to comply with each recommendation (Wilson et al., 2015).

In addition to suggesting higher limits for concurrent cognitive, affective, and neural plasticity than is widely assumed, this research has broad implications. While the present investigation elicited considerable improvements within a non-clinical sample of adults, even more extensive changes may be possible with clinical samples that have greater room for improvement. The present findings have particular relevance for a network analysis approach to mental illness. Rather than view mental disorders as underlying conditions that exist independently of symptoms, a network analysis approach views disorders as systems of causally connected symptoms (Borsboom and Cramer, 2013). By this perspective, the constellation of interacting symptoms is not a consequence of the disorder but rather *is* the disorder itself. This approach suggests that therapeutic interventions should directly target interrelated symptoms and the causal relations between them. Simultaneously targeting a network of mutually reinforcing symptoms may be best achieved through a highly multifaceted intervention. Although there is precedent for clinical samples to receive highly intensive and multifaceted treatments (e.g., inpatient addiction programs), challenges of feasibility may limit the investigation and application of these approaches.

The large and rapid improvements that were observed in cognitive abilities, affect, and physiology suggest that similarly modeled interventions could be an effective complement to existing institutionalized educational systems, which already have nearly full-time access to students for more than a decade of their lives. Inpatient clinical settings may also benefit from structuring time that is not devoted to clinical activities—which is often self-directed and under-utilized—toward a variety of evidence-based activities that would support comprehensive personal development. This logic applies broadly to a range of public and private institutions, including retirement communities, unemployment services, homeless shelters, and detention centers.

Although the improvements in cognition and well-being resulting from the present intervention are broad and substantial, they likely represent only a modest preview of what will ultimately be achieved through future interventions that draw on continual advances in science and technology. The true limits of cognitive, affective, and neural plasticity remain a mostly unexplored frontier of scientific understanding.

## Funding

This research was supported by the Institute of Education Sciences grant R305A110277 and the John Templeton Foundation grant 52071. The content does not necessarily reflect the position or policy of the U.S. government.

## Author Contributions

All authors contributed to the study design. MDM and KLM contributed to the intervention. MDM, BWM, and JWS performed the data analysis and interpretation. All authors contributed to the manuscript.

## Conflict of Interest Statement

The authors declare that the research was conducted in the absence of any commercial or financial relationships that could be construed as a potential conflict of interest.

## Footnotes

^1^http://surfer.nmr.mgh.harvard.edu/

^2^http://stnava.github.io/ANTs/

^3^http://www.nitrc.org/projects/artifact_detect

^4^http://icatb.sourceforge.net
